# Supplementary material for: Light-Triggered Cellular Delivery of Oligonucleotides
Source: Pharmaceutics. 2019 Feb 21;11(2):90. doi: 10.3390/pharmaceutics11020090 (PMC6410276; doi:10.3390/pharmaceutics11020090)
Supplement: Supplementary file 1 [file pharmaceutics-11-00090-s001.pdf]

pharmaceutics

Supplementary materials: Light-Triggered Cellular Delivery of Oligonucleotides

Leena-Stiina Kontturi, Joep van den Dikkenberg, Arto Urtti, Wim E. Hennink and Enrico Mastrobattista

Table S1. Analysis file exported from Columbus software used to determine the transfection percentages of HeLa S3 IVS2-654 EGFP cells.

Image Analysis Sequence Used			
Input Image	**Stack Processing :** Maximum projection **Flatfield Correction :** None		
Find Nuclei	**Channel :** BP445/45 **ROI :** None	**Method :** C Common Threshold : 0.05 Area : > 30 µm² Split Factor : 7 Individual Threshold : 0.4 Contrast : > 0.1	Output Population : Cells
Find Cytoplasm	**Channel :** BP525/50 **Nuclei :** Cells	**Method :** A Individual Threshold : 0.15	
Calculate Intensity Properties	**Channel :** BP525/50 **Population :** Cells **Region :** Cytoplasm	**Method :** Standard Mean	Output Properties : Intensity Cytoplasm BP525/50
Select Population	**Population :** Cells	**Method :** Filter by Property Intensity Cytoplasm BP525/50 Mean > 190	Output Population : Cells Selected
Define Results	**Method :** Formula Output Formula : a/b Population Type : Objects Variable A : Cells Selected - Number of Objects Variable B : Cells - Number of Objects Output Name : Number positive<br><br>**Population : Cells :** None Population : Cells Selected : None		

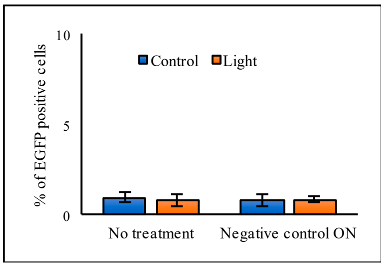

Figure S2. Transfection efficacy of the negative control oligonucleotide in HeLa S3 IVS2-654 EGFP cells. The cells were incubated in growth medium (no treatment) or with 1.4 mM ICG liposomes encapsulated with a negative control oligonucleotide (antisense oligonucleotide directed to position 705 at the EGFP cDNA) having 1/50 ICG/lipid ratio. After incubation, the cells were exposed to 808 nm light (370 mW/cm² for 2 min). The columns represent average values of EGFP positive cells (n = 3) with error bars as standard deviation. The threshold value to determine the EGFP positive and negative cells (transfected and non-transfected cells, respectively) was set based on the EGFP intensity of non-treated cells (less than 1% of EGFP positive cells for the non-treated cells).

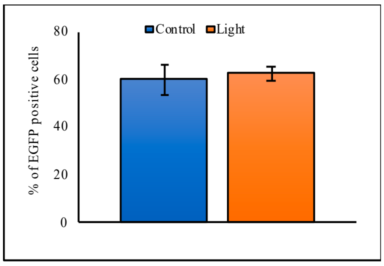

Figure S3. Effect of light exposure on transfection efficacy of Lipofectamine-SSO complexes in HeLa S3 IVS2-654 EGFP cells. The transfection was carried out by following the manufacturer's protocol with the recommended SSO concentration of 33 nM (0.25 µl Lipofectamine and 5 pmol SSO/well of a 96-well plate). The cells were incubated with the complexes for 4 hours, after which the medium was changed and the cells were exposed to 808 nm light with the intensity of 370 mW/cm² for 2 min. Control cells were shielded from the light. The columns represent average values of EGFP positive cells (n = 3) with error bars as standard deviation. Cells without treatment had less than 1% of EGFP positive cells.

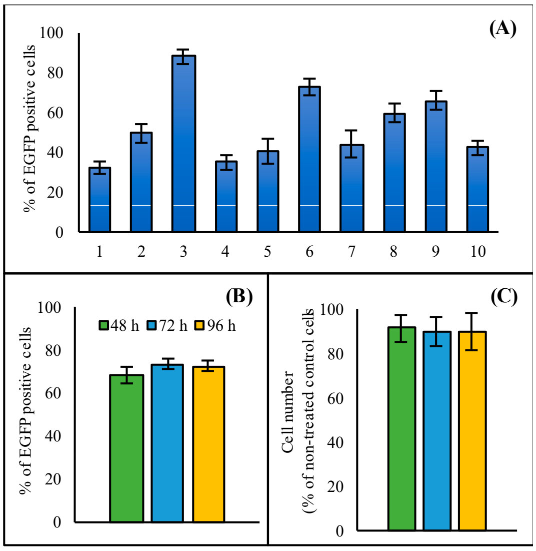

Figure S4. Transfection efficacy and cytotoxicity of Lipofectamine-SSO complexes in HeLa S3 IVS2-654 EGFP cells. (A) Transfection efficacy from 10 independent experiments showing the variability between experiments. The columns represent transfection

percentages from each experiment (average values, n = 3). (B) Transfection efficacy and (C) cell number measured at 48, 72 and 96 h after transfection. The percentage of transfected cells or cell growth do not change by time. The transfection was carried out by following the manufacturer's protocol with the recommended SSO concentration of 33 nM (0.25 µl Lipofectamine and 5 pmol SSO/well of a 96-well plate). Error bars represent standard deviations.

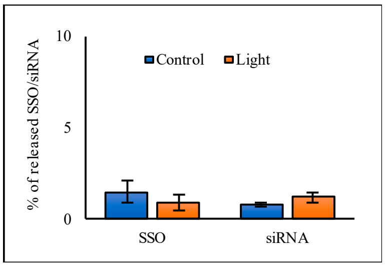

Figure S5. Light triggered release of SSO and siRNA from temperature sensitive liposomes without ICG. The liposomes were prepared as described in section 2.2. ("Liposomes preparation") in Materials and methods, but without ICG. The liposomes were kept at 37 °C and exposed to 808 nm light of 370 mW/cm^2 intensity for 2 min (light triggered samples, orange columns) or shielded from the light (control samples, blue columns). The columns represent average values of released SSO or siRNA (n = 3) with error bars as standard deviations.

Table S2. Intensity outputs of the laser with different power settings. Intensities were measured with a P-9710-1 optometer with RCH-102-2 custom made detector head (Te Lintelo Systems BV, Zevenaar, The Netherlands).

Power (W)	Intensity (mW/cm^2)
1	0–0.5
2	370
3	960
4	1500
5	2000
6	2600
7	3000
8	3600
9	4000
10	4300
